# TREM1 regulates antifungal immune responses in invasive pulmonary aspergillosis

**DOI:** 10.1080/21505594.2021.1879471

**Published:** 2021-02-02

**Authors:** L Bernal-Martínez, SM Gonçalves, B de Andres, C Cunha, I Gonzalez Jimenez, K Lagrou, E Mellado, ML Gaspar, JA Maertens, A Carvalho, L Alcazar-Fuoli

**Affiliations:** a Mycology Reference Laboratory, National Centre for Microbiology, Instituto De Salud Carlos III, Madrid, Spain; b Spanish Network for the Research in Infectious Diseases (REIPI), Instituto de Salud Carlos III, Madrid, Spain; c Life and Health Sciences Research Institute (ICVS), School of Medicine, University of Minho, Braga, Portugal; d ICVS/3B’s-PT Government Associate Laboratory, Braga/Guimarães, Portugal; e Department of Immunology, National Centre for Microbiology, Instituto De Salud Carlos III, Madrid, Spain; f Department of Microbiology, Immunology, and Transplantation, Laboratory of Clinical Bacteriology and Mycology, KU Leuven, Leuven, Belgium; g Department of Laboratory Medicine and National Reference Center for Medical Mycology, University Hospitals Leuven, Leuven, Belgium; h Department of Haematology, University Hospitals Leuven, Leuven, Belgium

**Keywords:** TREM1, *aspergillus fumigatus*, diagnostic biomarkers and fungal immune response

## Abstract

Pattern recognition receptors (PRRs) are responsible for *Aspergillus fumigatus* recognition by innate immunity and its subsequent immune signaling. The triggering receptor expressed on myeloid cells 1 (TREM1) is a recently characterized pro-inflammatory receptor constitutively expressed on the surface of neutrophils and macrophages. A soluble form (sTREM1) of this protein that can be detected in human body fluids has been identified. Here we investigated the role of TREM1 during invasive pulmonary aspergillosis (IPA). IPA patients displayed significantly higher levels of sTREM1 in bronchoalveolar lavages when compared to control patients. Functional analysis in TREM1 showed that the levels of sTREM1 and TREM1 pathway-related cytokines were influenced by single nucleotide polymorphisms in TREM1. In addition, we confirmed a role of TREM1 on antifungal host defense against *A. fumigatus* in a murine model of IPA. TREM1 deficiency increased susceptibility to infection in the immunosuppressed murine host. Deletion of TREM1 showed delayed innate and adaptive immune responses and impaired pro-inflammatory cytokine responses. The absence of TREM1 in primary macrophages attenuated the TLR signaling by altering the expression of both receptor and effector proteins that are critical to the response against *A. fumigatus*. In this study, and for the first time, we demonstrate the key role for the TREM1 receptor pathway during IPA.

## Introduction

Invasive Pulmonary Aspergillosis (IPA) is a severe disease that can develop in immunocompromised hosts, such as individuals with neutropenia, those treated for hematological malignancies, and recipients of hematopoietic stem cell (HSCT) or solid organ transplantation (SOT) [1–3]. Despite improvements in antifungal therapy and the availability of better diagnostic tools, the detection, definitive diagnosis, and treatment of this disease remains challenging.

IPA usually starts in the lungs via the inhalation of airborne conidia. There, innate immunity is mediated by neutrophils, monocytes, and alveolar macrophages, which are the first line of defense against *A. fumigatus* and responsible for local inflammatory responses against this pathogen [4,5]. The recognition of *A. fumigatus* by macrophages is performed by pattern recognition receptors, which include C-type lectin and toll-like receptors (TLRs) that recognize specific fungal components [6–8]. Over the last decade, an additional family of evolutionary conserved innate immune receptors has been identified: the triggering receptors expressed on myeloid cells (TREMs) [9]. Among them, TREM1 is the best-characterized member of the TREM family of receptors. TREM1, belonging to the Ig superfamily, is a cell-surface activating receptor with a single extracellular V-type Ig-like domain, a transmembrane region containing charged lysine residues, and a short cytoplasmic tail lacking signaling motifs. Receptor domains of TREM1 include both activating and inhibitory isoforms whose ligands are unknown [10].

TREM1 is constitutively expressed by neutrophils and macrophages and its expression is further up-regulated by microbial components [11]. Although the precise role of TREM1 has not yet been clearly defined, it is well documented that TREM1 amplifies TLR4 and TLR2 inflammatory signaling [12,13]. In addition, TREM1 can be secreted as a soluble form (sTREM1), and therefore it has been utilized as a diagnostic marker for inflammatory processes including infections. For instance, the presence of sTREM1 in samples from bronchoalveolar lavage (BAL) fluid from mechanically ventilated patients has been shown to be a good indicator of infectious pneumonia [14].

Compared to sepsis and bacterial pneumonia, studies evaluating the role of TREM1 in aspergillosis are relatively limited. Increased TREM1 expression is observed in monocyte-derived macrophages from patients with chronic pulmonary aspergillosis, while reduced TREM1 expression is observed in individuals suffering from allergic bronchopulmonary aspergillosis [15]. In addition, TREM1 has been shown to modulate the immune response directed against *A. fumigatus* during experimental fungal asthma [16] and in infected rat corneal epithelium [17].

In the present study, we aimed to characterize the role of TREM1 in IPA. We assessed sTREM1 levels in clinical samples of IPA patients compared to controls and we tested for TREM1 functionality by assessing whether individual genetic variation in TREM1 had an impact on the detected levels of sTREM1 and other cytokine-driven immune responses against *A. fumigatus*. In addition to gain a deeper understanding of the role of TREM1, we compared the course of *A. fumigatus* infections and immune responses in TREM1-deficient (*Trem*1^−/−^) and wild-type (*Trem*1^+/+^) mice.

## Material and methods

### Patients and sample collection

Bronchoalveolar lavage (BAL) samples were collected from hospitalized adult patients (≥18 years of age) during routine diagnostic workup following suspicion of infection at the Leuven University Hospitals, Leuven, Belgium. Fifty cases of “probable” or “proven” IA were identified according to the 2008 standard criteria of the European Organization for Research and Treatment of Cancer/Mycology Study Group (EORTC/MSG) [18]. BAL specimens were collected as described before [19]. All samples were stored at −80°C until use. The demographic and clinical characteristics of the patients enrolled in this study are described in Table 1.

**Table 1.** Baseline characteristics of patients enrolled in the study

**Variables**	**IPA** **(n = 50)**	**No IPA** **(n = 48)**	**P value**
**Age, no (%)**			
≤50 years	9 (18.0)	13 (27.1)	0.34
>50 years	41 (82.0)	35 (72.9)	
**Gender, no (%)**			
Female	23 (46.0)	21 (43.8)	0.84
Male	27 (54.0)	27 (56.2)	
**Underlying disease, no (%)**			
SOT*	12 (24.0)	20 (41.7)	0.22
Acute leukemia	9 (18.0)	10 (20.8)	
Allogeneic HSCT	9 (18.0)	6 (12.5)	
Chronic lymphoproliferative diseases	7 (14.0)	6 (12.5)	
Influenza A (H1N1)	6 (12.0)	0 (0.0)	
Chronic lung diseases	2 (4.0)	1 (2.1)	
Solid tumors	2 (4.0)	1 (2.1)	
Other	3 (6.0)	4 (8.3)	
**Severe neutropenia, no (%)†**	15 (30.0)	5 (10.4)	0.023
**BAL cell counts, mean (range)‡**			
Neutrophils	5.5 (0.0–30.3)	4.5 (0.1–17.3)	0.30
Lymphocytes	4.2 (0–175.7)	1.8 (0.2–17.4)	<0.001
**GMI, mean (range)**	5.1 (1.0–6.9)	0.11 (0.1–0.2)	<0.001
**Other pathogens detected in BAL, no (%)** Virus Bacteria Fungi	25 (50.0) 10 (20.0) 8 (16.0)	16 (33.3) 8 (16.7) 5 (10.4)	0.92

SOT, solid organ transplantation; HSCT, hematopoietic stem-cell transplantation; BAL, bronchoalveolar lavage; GMI, galactomannan index; P values were calculated by Fisher’s exact probability t-test for categorical variables, or by Student’s t-test or Mann–Whitney U test for continuous variables. *The study included 32 patients who received a SOT from lung (n = 26), kidney (n = 2), and liver (n = 2). Among those, 12 were diagnosed with IPA (lung, n = 8; kidney, n = 2; and liver, n = 2). †Severe neutropenia was defined as ≤0.5 × 10^9^ cells/L. ‡Cell counts in BAL were expressed as the number ×10^3^ cells/µL.

## Analysis of sTREM1 and cytokines in human BAL

sTREM1 levels and selected cytokines were quantified in BALs using customized Human Premixed Multi-Analyte Kits (R&D Systems, MN, USA). All determinations were performed in duplicates, and concentrations were reported in pg/mL.

## Single nucleotide polymorphism (SNP) selection and genotyping

The rs2234237 and rs2234246 SNPs in TREM1 were studied. Genomic DNA was isolated from BAL samples using QIAcube automated system (Qiagen, Hilden, Germany). Genotyping was performed using KASPar assays (LGC Genomics, Hertfordshire, UK) in an Applied Biosystems 7500 Fast Real-Time PCR system (Thermo Fisher Scientific, MA, USA), according to the manufacturer’s instructions. Mean call rate for the SNPs was >98%. Quality control for the genotyping results was achieved with negative controls and randomly selected samples with known genotypes.

## *Aspergillus fumigatus* growth conditions

The ATCC46645 strain was used throughout this work. *Aspergillus* was grown for 2 to 3 days at 37°C on potato dextrose agar slants. For all experiments, *A. fumigatus* spores were harvested and prepared as previously described [20].

## Murine infections

Eight weeks-old male TREM1-deficient (*Trem*1*^−^*^/−^) and wild type (WT) (*Trem*1^+/+^) mice, raised on C57BL/6 N background, were used. Mice were bred in house at the National Center for Microbiology. Immunosuppression was induced with 150 mg kg−1 cyclophosphamide (Pras‐Farma, Barcelona, Spain) administered intraperitoneally, and 112 mg kg−1 of Cortisone 21‐acetate (Sigma, C‐3130) administered subcutaneously, both on day −3 and −1. On day 0, the animals were anesthetized intramuscularly with 0.1 ml of a mixture of KetolarR (ketamine 50 mg ml−1; Pfizer) and RompumR (xylazin chlorohydrate 2%; Bayer) at final concentrations of 12.5 and 2 mg ml−1, respectively, followed by the intranasal inoculation of 30 μl saline suspension containing 2 × 10^7^ conidia per mouse. After infection, cyclophosphamide (150 mg kg−1) was used every three days until completion of the experiment.

Mice were weighed every 24 hours from the day of infection and visual inspection was made twice daily. Mice dying of fungal challenge were recorded daily for survival analysis. Depending on the experiment, mice were culled and whole lungs were collected after two, three, or five days post-infection (dpi). We used 10 to 12 mice per condition for survival, three mice per day for immune response and histology analyses. Uninfected animals were included as controls in all experiments. At least two independent murine infections were performed.

## Histology analyses

Mice were culled and whole lungs were collected from 2 dpi. Lungs were immediately fixed in 10% neutral-buffered formalin. After fixation, lung samples were then treated with xylene and embedded in paraffin. Five-micron tissue sections were stained with hematoxylin and eosin (HE) and sections were examined with a Leica DMI 3000B microscope.

## Flow cytometry

Whole lung cell suspensions from *Trem*1*^+/+^* and *Trem*1*^−/−^* infected mice at different times were prepared, counted, and stained with appropriate mAbs. Details on methods for flow cytometry analyses and antibodies are described in the Supplementary Methods. Neutrophils were identified by the expression of GR-1 ^hi^CD11b^hi^ and monocytes as GR-1^−^CD11b^+^. To confirm an adequate separation between neutrophils and monocytes, the expression of Ly6C, F4/80 and CD11c was traced in gated GR-1 ^hi^CD11b^hi^, being Ly6C^lo^, F4/80^lo^, CD11c^lo^ as described before (data not shown [21,22]).

## Analysis of sTREM1 and cytokines in lysates of homogenized murine lungs

sTREM1 concentrations were measured with the TREM1 DuoSet (R&D) according to the manufacturer’s instructions.

Cytokines were determined in 50-μl samples of whole lung homogenates using customized Mouse Premixed Multi-Analyte Kits (R&D Systems, MN, USA) and the Bio-Plex platform (Bio-Rad Laboratories, Hercules, CA).

## Isolation of murine macrophages from the peritoneal cavities

Primary macrophages were isolated from the peritoneal cavities of mice as described before [23]. After centrifugation of the cell suspension, primary macrophages were counted using a hemocytometer. A suspension of 5 × 10^5^ cells/ml in feeding medium RPMI 1640 w/o L-glutamine supplemented with 10% heat-inactivated fetal bovine serum (FBS) and L-Glutamine–Penicillin–Streptomycin was prepared, and 1 ml per well was added to 24 well tissue culture plates and incubated overnight at 37°C in a 5% CO2-enriched atmosphere. The following day the medium was removed, and 1 ml of fresh feeding medium containing *A. fumigatus* spores at a cell density of 5 × 10^5^ cells/ml was added to each well. Cell stimulation was carried out for 1 or 2 and 6 hours at 37°C in the presence of 5% CO2. Negative control included non-infected cells.

## Internalization of *Aspergillus fumigatus* conidia

Peritoneal mice macrophages were obtained and stimulated with *A. fumigatus* conidia as described before. Conidia were added to the macrophages and after 1 h of incubation at 37°C, all content of the wells was discarded, wells were washed three times with PBS and the host cells were lysed with water. Content wells were collected and plated in serial dilution on Sabouraud agar in order to obtain conidia associated with cells. Wells without cells were included and after the incubation period, all the content was collected and plated in serial dilution on Sabouraud agar. CFUs (colony-forming units) were counted after 24 h at 37°C. Conidia associated with cells were compared to the inoculum of conidia in wells without cells to calculate the percentage phagocytosis.

## RNA extraction and quantitative PCR (qPCR)

After induction with *A. fumigatus* during 2 and 6 hours, the culture medium was removed and wells were washed twice using PBS. RNA was isolated from TRIzol (Invitrogen Life Technologies) suspended samples using RNeasy Mini kit from Qiagen. The purified RNA was subsequently used for cDNA preparation using the ImProm-IITM Reverse Transcription System kit (Promega). Primers used for qPCR are described in Supplementary table 2. The analysis of gene expression was performed using quantitative PCR (qPCR) SensiMix™ SYBR® Hi-ROX Kit (Roche Diagnostic) and performed in a CFX96 system (Bio-Rad). The actin gene was used as housekeeping and fold change in gene expression was calculated over non-infected cells using the 2^−ΔΔCT^ threshold cycle (CT) method [24].

## Statistical analyses

All data were analyzed with GraphPad Prism software. Unpaired t-tests were used to determine statistically significant differences between experimental groups in which p values <0.05 were considered statistically significant.

The area under the receiving operating characteristic curve (AUCROC) was used to evaluate the ability of TREM1 to diagnose IPA. Youden index was computed to determine the sTREM1 cutoff level with the highest discriminatory power.

Kaplan-Meier survival curves were analyzed by using a log-rank (Mantel-Cox) test for significance. A p-value <0.01 was considered statistically significant.

## Ethics statement

Study approval was obtained for the use of human samples from the Ethics Subcommittee for Life and Health Sciences of the University of Minho, Portugal (protocols 125/014 and 126/014), and the Ethics Committee of the University Hospitals of Leuven, Belgium.

Murine infections were performed in dedicated facilities of the National Center for Microbiology. The project was ethically approved by the Animal Welfare Committee of the Instituto de Salud Carlos III and performed under the approved Project License PROEX 324/16.

## Results

### TREM1 levels in BAL samples are associated with probable and proven IPA

**Figure 1.**
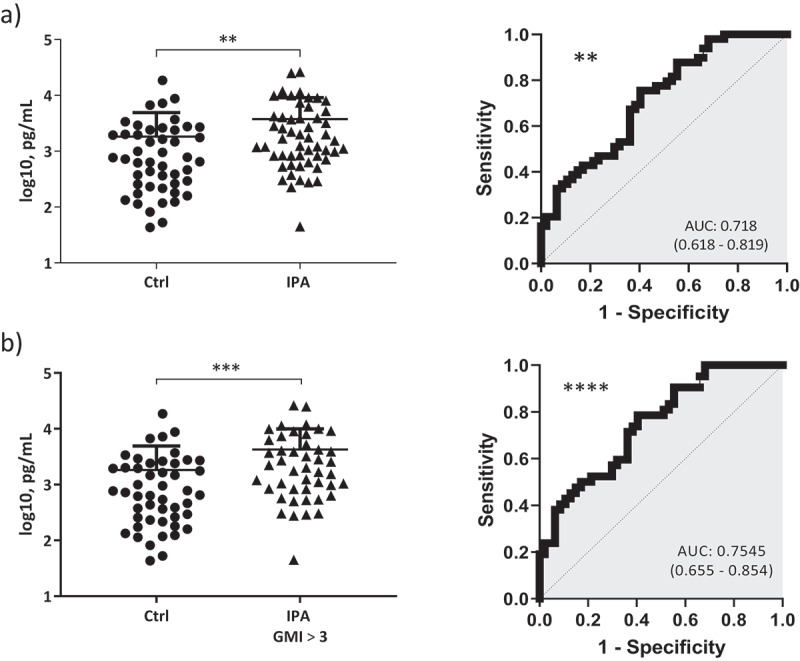
a) Left, quantification of sTREM1 levels in BAL samples from patients with IPA (cases, n = 50) compared to controls (n = 48). sTREM1 levels were expressed as log10 pg/mL. Data are presented as scatterplots representing individual data points and a line indicating the mean value with SD. Right, receiver operating characteristic (ROC) curves used to evaluate the ability of sTREM1 to diagnose invasive pulmonary aspergillosis (IPA). B) Left, quantification of sTREM1 levels in BAL samples from patients with IPA and high GMI > 3.0 compared to controls. TREM1 levels were expressed as log10 pg/mL. Data are presented as scatterplots representing individual data points and a line indicating the mean value with Sd. Right, receiver operating characteristic (ROC) curves used to evaluate the ability of sTREM1 to diagnose IPA. p-values from sTREM1 levels comparisons were calculated using the Mann–Whitney U test. ****, p < 0.0001, ***, p < 0.001; **, p < 0.01

To investigate the role of TREM1 in IPA, we compared the levels of TREM1 in BAL fluids from patients diagnosed with IPA and matched controls (non-IPA patients). A total of 98 subjects, including 50 cases of “probable” or “proven” IA according to the EORTC/MSG criteria and 48 controls with no *A. fumigatus* infection were included in this study (Table 1). The distribution and comparison of BAL biomarker levels between different groups of patients are shown in Figure 1a. IPA patients showed significantly higher levels of sTREM1 than control patients. To corroborate its utility for the diagnosis of IPA, we analyzed the AUCROC for sTREM1 (Figure 1a). sTREM1 demonstrated reasonable sensitivity and specificity (AUCROC = 0.718; 95% CI, 0.618–0.819; p = 0.002) with a cutoff level ≥ 800.8 pg/mL calculated using Youdens index.

Detection of galactomannan in BAL samples has been described as a sensitive test for diagnosing of IPA [25], therefore the association between sTREM1 levels in BAL and galactomannan was analyzed. We categorized the patients into subgroups according to the galactomannan index (GMI): GMI <0.5 (identifying controls), and high GMI ≥3.0 (associated with a diagnosis with 100% specificity) [26]. We found that patients with higher levels of galactomannan (GMI ≥3.0) also displayed significantly elevated concentrations of sTREM1 compared to GMI-negative controls (GMI <0.5) which increased the statistical significance to p < 0.001 (p = 0.0006). Likewise, the statistical significance of AUCROC was also increased (AUCROC = 0.7545; 95% CI, 0.655–0.854; P < 0.0001) (Figure 1b).

## TREM1 SNPs can regulate sTREM1 levels and inflammatory responses in IPA patients

To examine the functionality of TREM1 in the context of IPA, we investigated whether SNPs in TREM1 could differentially regulate sTREM1 levels in BAL samples from patients with IPA and matched controls. We studied the rs2234237 SNP, a nonsynonymous variation (Ser25Thr) located in exon-2 that may influence the biologic function of TREM1 [27], and the rs2234246 SNP, located at gene promoter and described to regulate TREM1 expression levels that results in differential plasma levels of proteins such as L-selectin [28].

**Figure 2.**
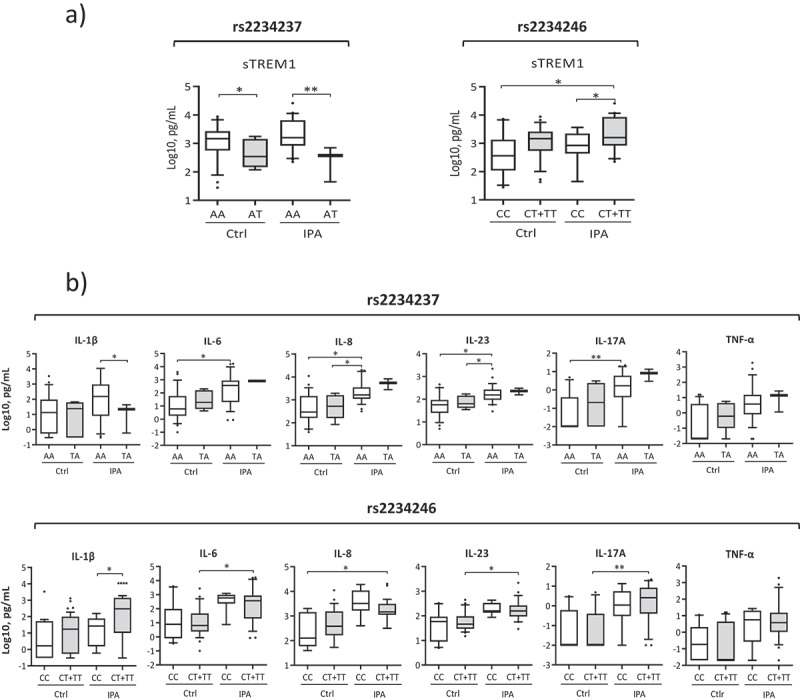
a) sTREM1 levels measured in BAL samples from IPA and control (Ctrl) patients and stratified based on TREM1 genotypes of gene polymorphisms (rs2234237 and rs2234246). b) Cytokines levels measured in BAL samples from IPA and control patients and stratified based on TREM1 genotypes (rs2234237, rs2234246). Data are presented as box plots, with the bottom and top of the box indicating the first quartile to the third quartile, respectively. The bar within the box indicate the median value and the whiskers above and below the box show the 10th and the 90th percentiles. The dots indicate outliers. p-values were calculated using the Welch’s t-test. **, p < 0.01; *, p < 0.05

We found that individuals harboring the AA genotype at rs2234237 showed higher levels of sTREM1 when compared to the AT genotype. Statistical differences were increased among IPA patients. The T allele at rs2234246 was associated with increased amounts of sTREM1 compared to the homozygous CC genotype in IPA patients (Figure 2a). Recent studies identified IL-1β, IL-6, IL-8, IL-17A, IL-23, and TNFα as alveolar cytokines that were significantly increased in BAL samples of IPA patients [19]. Since TREM1 acts as an amplifier of the immune response and its stimulation promotes the release of several pro-inflammatory cytokines [29], we investigated the associations between the significance in the above SNPs and the levels of those cytokines with an important role in the lung environment. We found that IPA patients harboring the AA genotype at rs2234237 and individuals having the T allele for rs2234246 displayed differential levels of IL-1β (Figure 2b). Interestingly, genotype-specific differences were observed, with control and IPA patients displaying differential concentrations of most studied cytokines (Figure 2b).

## Course of IPA in immunocompetent or immunosuppressed *Trem*1^−/−^ and *Trem*1^+/+^ mice

**Figure 3.**
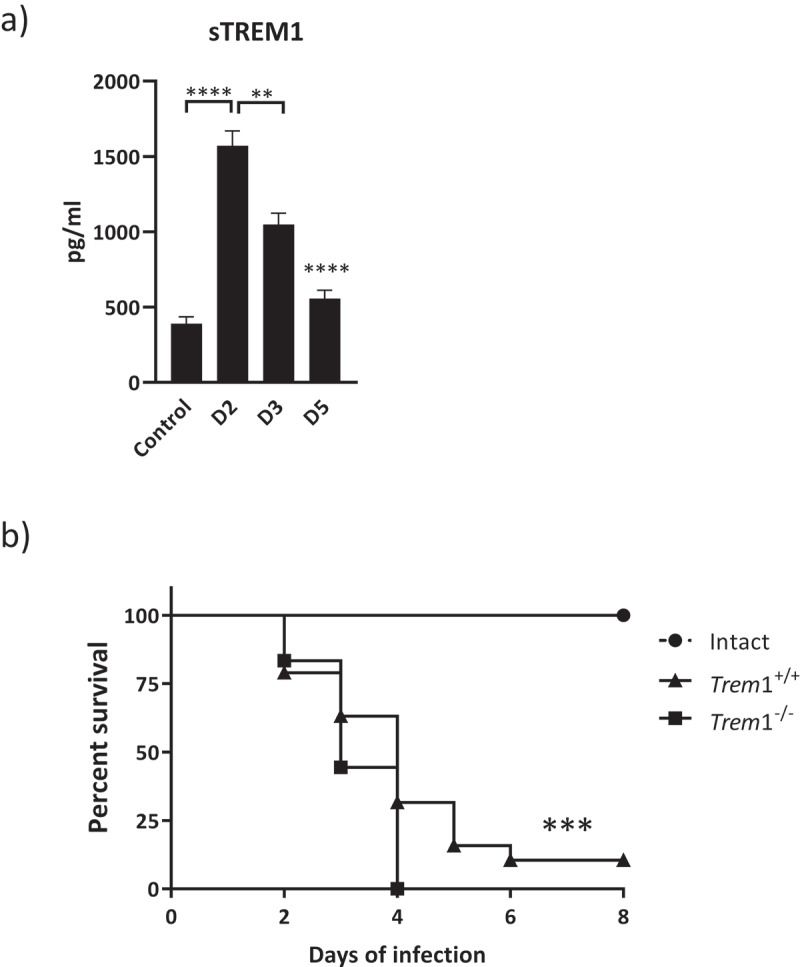
a) sTREM1 concentrations in lung homogenates from WT mice during *A. fumigatus* infection at different times (D2, 2 days; D3, 3 days; D5, 5 days). Values are represented as bar plots with mean ± SEM. p-values were calculated using the Mann–Whitney U test. ****, p < 0.0001; **, p < 0.01. b) Comparison of survival rate of WT mice (*Trem*1^+/+^; black lines with triangles) and TREM1-deficient mice (*Trem*1^−/−^; black lines with squares) to IPA. Intact mice (black lines with circle). Data represent the cumulative data of two separate experiments. The p-value of Kaplan–Meier curves comparison of immunosuppressed mice was determined with the use of the log-rank test. ***, p < 0.001

TREM1 gene expression induction, together with other genes of the pathway, was first seen in a previous transcriptomic analysis of murine lungs infected with *A. fumigatus* (Alcazar-Fuoli L., personal communication, Figure S1). To confirm these findings and to correlate increasing sTREM1 levels in response to *A. fumigatus* infection in mice, we assessed sTREM1 levels in lung homogenates of WT mice (Figure 3a). We observed that those levels were significantly increased after infection and then decreased gradually.

To further investigate the role of TREM1 in the context of IPA, the impact of TREM1 deficiency on susceptibility to aspergillosis was evaluated in experimental murine models. Immunocompetent (intact) and immunosuppressed *Trem*1^−/−^ and *Trem*1^+/+^ mice were inoculated with viable *A. fumigatus* conidia.

All intact *Trem*1^+/+^ and *Trem*1^−/−^ mice survived to the *A. fumigatus* challenge (Figure 3b). As expected, immunosuppression resulted in enhanced susceptibility in both genetic backgrounds, however, immunosuppressed *Trem*1^−/−^ mice showed earlier and more evident symptoms typical of the in vivo mouse model of aspergillosis such as hunching, head tilting, and circling [30] compared to *Trem*1^+/+^ mice. Under immunosuppressed conditions, *Trem*1^−/−^ mice showed a reduced survival that resulted statistically significant compared to *Trem*1^+/+^ mice (Figure 3b). About a 10% of *Trem*1^+/+^ mice survived the infection while none of the *Trem*1^−/−^ survived. Median survival for *Trem*1^−/−^ was 3 days while it was 4 days for the *Trem*1^+/+^ mice.

## TREM1 deficiency showed a defective immune cell recruitment to the lungs

**Figure 4.**
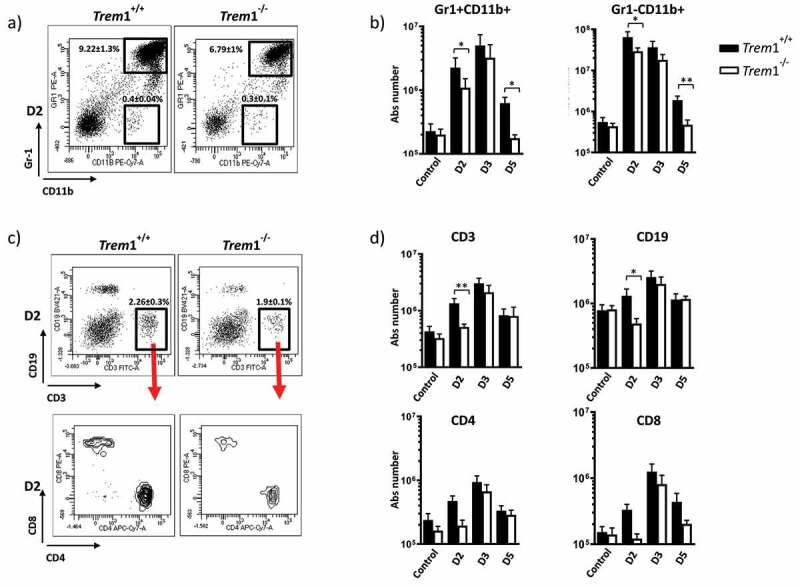
Whole lung cell suspensions from *Trem*1^+/+^ and *Trem1*^−/−^ infected mice at different times (D2, 2 days; D3, 3 days; D5, 5 days) and control mice were prepared, counted and stained with the mAbs indicated below for flow cytometry analyses (see Materials and Methods) using anti-CD11b-PECy7 and anti-Gr-1-PE (myeloid cells), anti-CD3-FITC, anti-CD4-APCCy7 and anti-CD8-PE (T cells), anti-CD19-Violet 421 (B cells). Viable cells were calculated on the basis of LIVE/DEAD violet-510 exclusion. Fluorescence scales are logarithmic. a) Representative dot plots of the myeloid cell populations at d2 pi in the myeloid gate to discriminate granulocytes (Gr-1+ CD11b+) from monocytes (Gr-1-CD11b+). b) Bar graphs represent total number of lung myeloid cells recovered from *Trem*1^+/+^ (black bars) and *Trem*1^−/−^ (white bars) from uninfected mice and after infection with *A. fumigatus* at different days post-infection. Calculations of the total number were performed from the frequencies obtained of each population and referred to the lung weight/total animal weight. c) Representative dot plots of the lymphoid cell populations at d2 pi from lymphoid gating. Arrows indicate gating on CD3+ cells in order to study CD4+ and CD8+ cells. d) Bar graphs represent total number of lung lymphoid cells from both strain mice at indicated days after infection and uninfected mice calculated as described before. Data are mean ± SEM (n = 6). Comparisons were performed by using a two-tailed Student t-test. **, p < 0.01; *, p < 0.05

Immune cell responses and particularly myeloid cells are altered under the immunosuppressed conditions that are normally used in murine models of IPA [31]. For this reason and in order to see the intact role of TREM1 in response to *A. fumigatus* infection we used from now on a non-immunosuppressed infection model. Lung preparations from *Trem*1^−/−^ and *Trem*1^+/+^ mice were analyzed on 2, 3 and 5 dpi. Innate immune response was evaluated by the presence of granulocytes (Gr-1+ CD11b+) and monocytes (Gr-1-CD11b+) (Figure 4a, Figure 4b). *Aspergillus* instillation induced an important amount of both populations after 2 dpi. In the case of *Trem1*^−/-^ infected mice, granulocytes and monocytes were strongly diminished in comparison with infected WT mice. No significant differences were observed in alveolar macrophage numbers in either strains (data not shown). Lymphocyte recruitment was determined by the presence of CD3+ (T-lymphocytes), and CD4+, CD8+ and CD19+ (B-lymphocytes) (Figure 4c, Figure 4d). A rapid recruitment of lymphocytes was detected after 2 dpi on WT mice. In the case of *Trem*1^−/-^ infected mice, minimal levels of CD3+ and CD19+ cells were detected at 2 dpi and a delayed lymphocytic response was detected from 3 dpi.

## Lungs from TREM1-deficient mice have decreased histological evidence of inflammation

**Figure 5.**
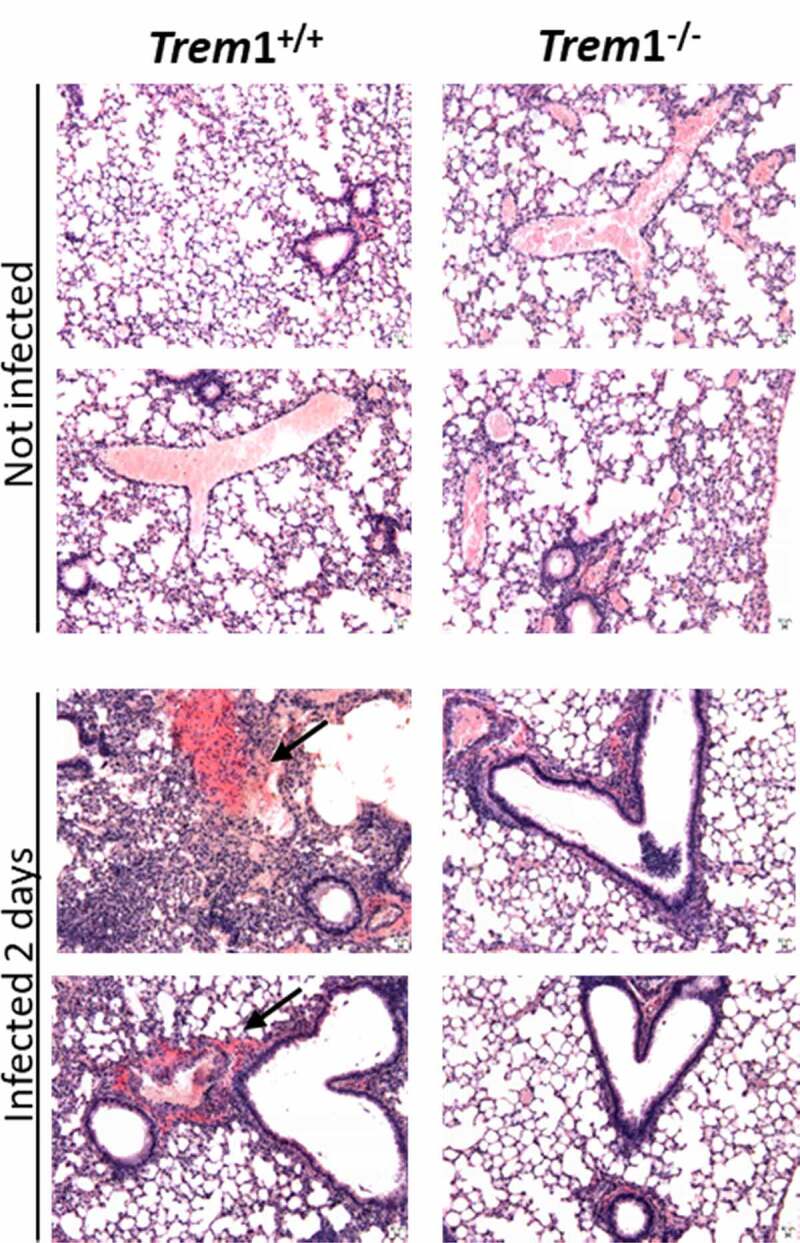
Histology of lung sections of *Trem*1^+/+^ and *Trem*1^−/−^ mice at day 2 post-infection versus non-infected controls, stained in HE. 10 x magnifications. Scale bars represent 10 μm

Histological examination of lungs harvested from mice at 2 dpi showed that TREM1-deficient animals had less evidence of inflammation when compared to WT animals (Figure 5). Lung histology of WT mice showed that infection with *A. fumigatus* promoted remarkable lung inflammatory responses, including significant infiltration of inflammatory cells and destruction of blood vessels (Figure 5, arrows).

## Deletion of TREM1 decreases cytokine responses against *A. fumigatus* in the murine lung

**Figure 6.**
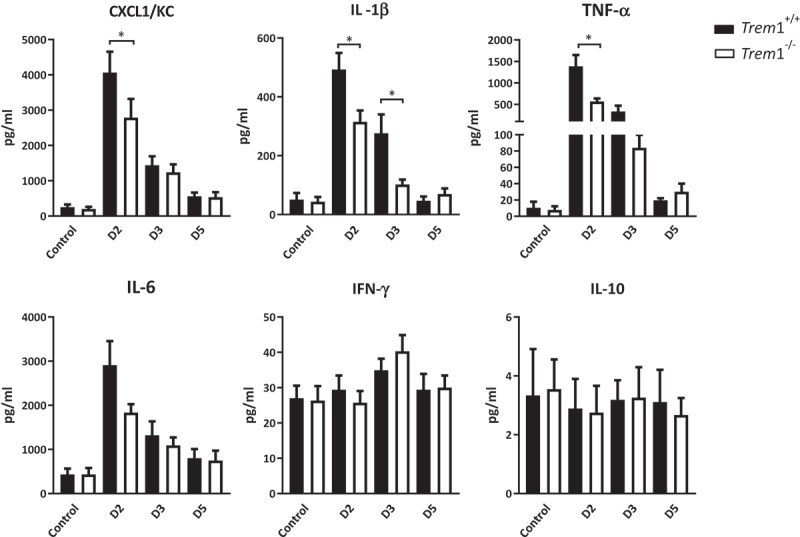
Chemokines and cytokines in TREM1-deficient (*Trem*1^−/−^) or wild-type (*Trem*1^+/+^) mice with IPA. Chemokines and cytokines were determined by Luminex assay in lung homogenates from uninfected mice or from mice at 2 (D2), 3 (D3) or 5 (D5) days after intranasal inoculation of A. fumigatus conidia. Values are represented as bar plots with mean ± SEM. P-values were calculated using the Mann–Whitney *U* test. **, p < 0.01; *, p < 0.05

To correlate the pattern of inflammatory pathology observed in infected mice with the production of chemokines and cytokines known to be associated with the TREM1 inflammatory pathway, the chemokine CXCL1/KC, the inflammatory cytokines IL-1β, TNF-α, and IL-6 as well as IL-10 and INF-γ, were measured in lung homogenates of *Trem*1^−/−^ and *Trem*1^+/+^ mice. In line with our previous results, an induction of inflammatory cytokines was observed with maximum amounts at 2 dpi that decreased between 3 and 5 dpi (Figure 6). Statistical differences were observed at day 2 between the WT and the *Trem*1^−/−^ mice for CXCL1 and TNF-α. Of interest, differences for IL-1β were also maintained at 3 dpi and no induction and differences between groups were obtained for IL-10 and IFN-γ (Figure 6).

## *In vitro* activity of peritoneal macrophages isolated from *Trem*1^−/−^ and *Trem*1^+/+^ mice

**Figure 7.**
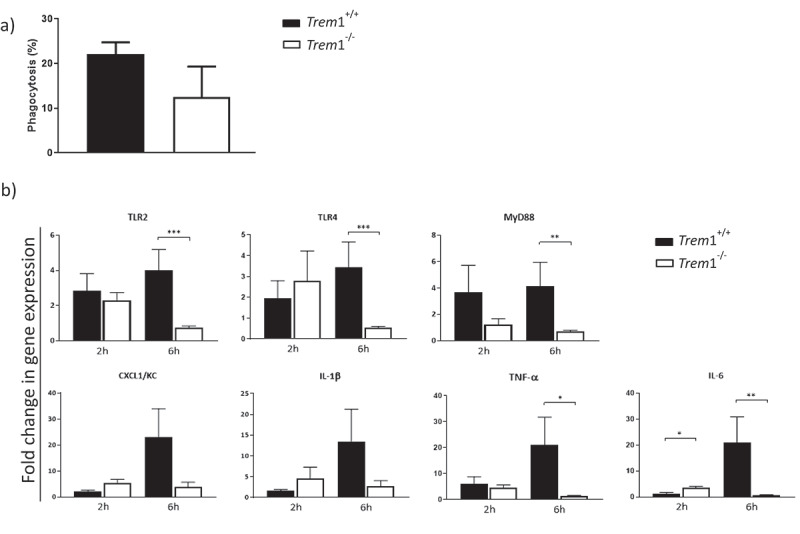
a) Percentage of phagocytosis of murine macrophages isolated from the peritoneal cavity of *Trem*1^−/−^ or *Trem*1^+/+^ mice and challenged with *A. fumigatus* for 1 hour. b) Fold change in gene expression levels of TLR2, TLR4, MyD88, CXCL1, IL1β, IL6 and TNFα in murine macrophages isolated from the peritoneal cavity of *Trem*1^−/−^ or *Trem*1^+/+^ mice and challenged with *A. fumigatus* for 2 and 6 hours. Values are represented as bar plots with mean ± SEM. p-values were calculated using the Mann–Whitney *U* test. **, p < 0.01; *, p < 0.05

Phagocytosis quantification showed a trend toward a decrease in the uptake of conidia by the macrophages isolated from *Trem*1^−/−^ mice (Figure 7a). To further investigate the synergy between TLRs and TREM1 in the immune responses against *A. fumigatus*, we analyzed the gene expression profile of primary murine macrophages within a background of complete TREM1 deficiency.

After *A. fumigatus* stimulation for 2 and 6 hours, the levels of gene expression of several genes related to TLR signaling and the TREM1 pathway inflammatory response were measured by real-time qPCR. Besides cytokines such as IL6, CXCL1, IL1β, and TNFα, we also evaluated gene expression of the receptors TLR2, TLR4, and the adaptor of MyD88.

In general gene expression was induced after 2 hours of infection in both genetic backgrounds. However after 6 hours of *A. fumigatus* exposure, TREM1-deficient macrophages demonstrated significantly lower gene expression of TLR2, TLR4, and MyD88 receptors as well as a significant gene expression reduction of IL6 and TNFα compared to the WT (Figure 7b).

## Discussion

TREM1 is an important regulator of innate immunity that promotes the amplification of inflammatory signals initiated by TLRs and NOD-like receptors [32]. TREM1 has been characterized as a major player in the pathophysiology of acute inflammatory diseases of different etiologies such as acute myocardial infarction, atherosclerosis, and infectious diseases [11,33,34]. TREM1 is an immune receptor expressed on innate immune cells, such as neutrophils, mature monocytes, macrophages [9], and platelets [35]. TREM1 is also expressed on lung alveolar macrophages, and this tissue-specific pattern of expression has suggested a potential role for this molecule in the activation of local inflammatory response during lung infections [36].

Besides the cell receptor, a soluble form of TREM1 (sTREM1) has been described [29]. sTREM1 may be produced from a splice variant or following the shedding of membrane-bound TREM-1 by metalloproteinase-mediated proteolytic cleavage [37]. The biological function of sTREM is still unknown but based on data of other soluble forms of membrane receptors, sTREM1 may act as a negative regulator of TREM1 signaling by neutralizing TREM1 ligands and thus interfere with the pro-inflammatory function of the TREM1 receptor [29]. sTREM1 can be detected in biological fluids during infection or inflammation, therefore, its relevance as a diagnostic biomarker is becoming important. There are several studies reporting that TREM1 plays a role in sepsis [38]. Plasma sTREM1 levels were positively correlated with high scores of severity and mortality [39]. Serum levels of sTREM1 have also been reported to predict bacteremia in patients with community-acquired pneumonia [40]. In a recent study, the association between serum sTREM1 levels and pulmonary tuberculosis (TB) showed that serum levels were significantly increased in TB patients and these higher serum levels correlated with disease severity and treatment outcomes [41].

A clear pathological hallmark of IPA is the pulmonary involvement which makes the BAL fluid examination a widely used procedure for the evaluation of patients with suspected aspergillosis. For instance, the detection of galactomannan, a polysaccharide present in the cell wall of *Aspergillus* species, in BAL fluids, has been advocated as a sensitive test for diagnosing IPA (26). In this study, we identified a specific role of sTREM1 in the clinical presentation of IPA. Patients with IPA had significantly higher levels of sTREM1 in BAL fluids when compared to control patients. Higher sTREM1 levels were also correlated with higher values of galactomannan. We could discard this finding as attributable to a general response to infection since events of viral, bacterial, and other fungi were diagnosed within the control group (Table 1). Considering the specific expression of TREM1 in the lung, we could suggest that the use of TREM1 alone or in combination with other biomarkers present in respiratory samples could improve our capacity to anticipate a more accurate diagnosis for IPA.

Functional analysis of SNPs in TREM1 has shown that levels of sTREM1 can be regulated by individual variability at genomic level with a potential impact on the progression and severity of the disease and patient outcome [28]. For instance, a clinically relevant association of the rs2234237 T-allele with higher levels of sTREM1 has been reported in mechanically ventilated burn patients and in sepsis [27,42]. TREM family receptors have been also suggested to regulate the cellular adhesion process and trans-endothelial migration [28]. In this context, the T allele at rs2234246 was associated with increased levels of sTREM1, affecting its expression levels, and with increased levels of soluble L-selectin which are thought to represent a homeostatic effort to limit excessive inflammation [28].

In our study, we stratified sTREM1 levels detected on the BAL of IPA and control patients according to TREM1 genotypes. We found changing levels of sTREM1 for rs2234237 and rs2234246 and we also found that TREM1 genotypes influenced the profile of proinflammatory cytokines. Based on the fact that in IPA higher levels of inflammatory mediators at the site of infection might indicate a more severe infection [19], we could hypothesize that TREM1 genotypes associated with an increase of sTREM1 levels and alteration of pro-inflammatory cytokines production in response to *Aspergillus* infection may impact disease progression that can ultimately affect patient outcomes.

We found that humans with pulmonary Aspergillosis manifested elevated levels of sTREM1 in BALs. Accordingly, mice infected with *A. fumigatus* showed increased lung sTREM1 levels (Figure 3a). In vivo, the role of TREM1 has been characterized in experimental murine models of microbial sepsis, infection with influenza virus, *Leishmania* major and other bacterial infections, in which suppression of TREM1 signaling conferred significant protection [43–45]. In these models, protection was associated with reduced severe inflammation and diminished expression of pro-inflammatory cytokines [46]. In the case of *A. fumigatus* infections, although reduced cytokine-driven inflammation can contribute to less damage in the lungs, impaired innate immune response and a lower capacity to mount early cytokine responses represent a primary risk factor for susceptibility, particularly in an immunosuppressed host [47]. In this sense, our study showed that, compared to WT mice, immunosuppressed *Trem*1^−/-^ mice were more susceptible to aspergillosis. We found that both granulocytes and monocytes were recruited after infection in the murine lungs although cell recruitment was affected in the TREM1–deficiency setting. Granulocytes and monocytes were diminished in *Trem*1^−/-^ infected mice compared to infected WT mice at 2 dpi. Fungal burden was checked in both groups (data not shown) to discard that the observed differences were not a consequence of differential fungal growth in the lungs. Furthermore, we showed that the difference in the amount of granulocytes and monocytes was still maintained after 5 dpi and they were below the initial values in the *Trem*1^−/-^ background. Since recruitment and activation of polymorphonuclear neutrophils and mononuclear cells into the lungs is a crucial event in the early phases of IPA [48], we could attribute an increased susceptibility of *Trem*1^−/-^ mice to an impaired pulmonary inflammation in response to *A. fumigatus* that impacted survival in the immunosuppressed host.

The innate immune response during *Aspergillus* infection triggers the development of an acquired immune response inducing the development of diverse CD4 T-cell responses that include T helper (Th) 1, Th2, Th17, and regulatory T (Treg) cells. In this sense, our results suggest an impaired immune response characterized by diminished recruitment of T-cells (CD3, CD4, and CD8) and B-cells (CD19) to the lungs of *Trem*1^−/-^ mice, especially at 2 dpi.

Histological visualization of lungs agreed with above-mentioned results and showed that infection with *A. fumigatus* produced lung inflammatory responses in both murine genetic backgrounds. However, WT mice showed a significant infiltration of inflammatory cells and it was possible to appreciate destruction of blood vessels. As described before [28], an important role of TREM1 has been reported in cellular adhesion and trans-endothelial migration of neutrophils and monocytes. The histological pattern of *Trem*1^−/-^ mice would correlate with an impaired recruitment of inflammatory cells in the lungs and an apparent decrease of tissue damage due to the absence of TREM1. Although we could hypothesize that suppression of the TREM1 pathway may be beneficial for the pathological processes associated with IPA, our results indicate a higher susceptibility to infection by *A. fumigatus*. This agrees with fact that a right balance between moderating excessive inflammation while preserving the capacity for microbial control is required for a successful resolution of *A. fumigatus* infections [7].

The recognition of *Aspergillus* conidia induces the expression of proinflammatory chemokines and cytokines [10;11]. In line with previous results, we observed that the complete deficiency of the receptor was associated with decreased *Aspergillus*-induced pro-inflammatory cytokine responses. The production of CXCL1, IL-1β, TNF, and IL-6 was decreased in the lungs of *Trem*1^−/-^ mice at early stages on the infection (2 dpi). Time course pro-inflammatory cytokine profiles in *Trem*1^+/+^ mice (Figure 6), followed the same pattern than the detection of sTREM1 (Figure 3a). While pro-inflammatory cytokines and sTREM1 levels decreased at 3 dpi, the number of immune cell was still increasing (Figure 4). These findings agree with the hypothesis that this soluble form can be neutralizing the production of cytokines [29] but it does not mediate a direct effect in the influx of immune cells.

The early control of microbe’s invasion primarily depends on professional phagocytes such as neutrophils and macrophages. In vitro studies have suggested that TREM1 may enhance phagocytosis [49]. In this study, we observed that the capacity of phagocytosis of *A. fumigatus* conidia was slightly reduced in the absence of TREM1. In addition, gene expression levels of TLR2, TLR4, the adaptor protein MyD88 as well as the pro-inflammatory cytokines IL-6, CXCL1, MCP1, IL1β, and TNFα, were quantified in order to corroborate the hypothesis of a synergistic effect between TREM1 and inflammatory reactions mediated by TLR4 and TLR2. Using murine macrophages isolated from *Trem*1^+/+^ and *Trem*1^−/-^ mice, we observed a decreased expression of all the studied genes in the absence of TREM1 after 6 hours of *A. fumigatus* challenge. These *in vitro* studies suggest a role for TREM1 in the recognition of *A. fumigatus* and support the role of TREM1 as an important modulator of the inflammatory response against *A. fumigatus*. From a mechanistic point of view, we could suggest that TREM1 acts as a supplementary molecule amplifying the immune response, but not initially inducing it, since its absence does not suppress completely the expression of TLR2 and TLR4. These receptors are important for the recruitment of polymorphonuclear cells to the lungs during IPA. The fact that the expression of the TLR‐MyD88 pathway is reduced in conditions of TREM1 deficiency will impact the response to *A. fumigatus* by restraining the release of cytokines and chemokines and less effective recruitment of immune cells to the lungs.

Collectively, our data provide evidence for a potential diagnostic value of sTREM1 in IPA. Initial findings on TREM1 functionality suggest that the impact of TREM1 SNPs in IPA should be further investigated. In addition, we showed a role of TREM1 on antifungal host defense against *A. fumigatus* in a murine model of IPA. TREM1 deficiency in mice, increased susceptibility to infection in an immunosuppressed murine model of IPA. Based on our findings, we can suggest that TREM1 deficiency mediates susceptibility against *Aspergillus* in mice due to a delayed innate and adaptive immune responses and impaired pro-inflammatory cytokine response.

## Supplementary Material

Supplemental Material
