# Supplementary material for: TREM1 regulates antifungal immune responses in invasive pulmonary aspergillosis
Source: Virulence. 2021 Feb 2;12(1):570–83. doi: 10.1080/21505594.2021.1879471 (PMC7872058; doi:10.1080/21505594.2021.1879471)
Supplement: Supplemental Material [file KVIR_A_1879471_SM6548.zip › Supplementary information/Suplementary table 2.docx]

| **Gene** | **Primer name** | **Sense** | **Sequence (5’-3’)** |
| --- | --- | --- | --- |
| **Actin** | Actin F  Actin R | Forward  Reverse | CGAGCACAGCTTCTTTGCAG  CCCATGGTGTCCGTTCTGA |
| **TREM1** | TREM1 F  TREM1 R | Forward  Reverse | ATAAATGGGACAGATGCT  TGACAATGAATAAGATGATGAA |
| **IL6** | IL6 F  IL6 R | Forward  Reverse | CCTGTCTATACCACTTCAC  AATCAGAATTGCCATTGC |
| **IL1β** | IL1β F  IL1β R | Forward  Reverse | CTCCGAGATGAACAACAA  TGATATTCTGTCCATTGAGG |
| **CXCL/KC1** | CXCL/KC1 F  CXCL1/KC R | Forward  Reverse | ATTCACCTCAAGAACATCC  GAGTGTGGCTATGACTTC |
| **MCP1** | MCP1 F  MCP1 R | Forward  Reverse | GCCAATCAGCCATCT  CCTCCATCAACCACTT |
| **MyD88** | MyD88 F  MyD88 R | Forward  Reverse | TTAGACCGTGAGGATATACT  GTTCTGCTGCTTACCTAA |
| **TLR2** | TLR2 F  TLR2 R | Forward  Reverse | CGAATCACAGTAGAGAACA  CGCTGAGGTCTAAGAATT |
| **TLR4** | TLR4 F  TLR4 R | Forward  Reverse | TTCAATCGCATAGAGACATC  TTCACATATACAAGCAACAGA |
| **TNFα** | TNFα F  TNFα R | Forward  Reverse | CCAAAGGGATGAGAAGTT  GAGAAGATGATCTGAGTGT |

**Supplementary Table 2**: Sequences of primers used for gene expression by Real Time qPCR.
