# Supplementary material for: TREM1 regulates antifungal immune responses in invasive pulmonary aspergillosis
Source: Virulence. 2021 Feb 2;12(1):570–83. doi: 10.1080/21505594.2021.1879471 (PMC7872058; doi:10.1080/21505594.2021.1879471)
Supplement: Supplemental Material [file KVIR_A_1879471_SM6548.zip › Supplementary information/Supplemental Material.docx]

**Supplemental Material**

**Figure S1.**

Heat-map of gene expression for genes involved in the TREM1 pathway. Coloration indicates magnitude of log2 ratio calculated from RNAseq data. Day 2 and day4 represents gene expression at two and four days’ post infection versus respectively uninfected animals. Black bars indicate that no differential gene expression was detected for a particular gene at the indicated time point.

**Supplementary methods.** Flow cytometry
