# Supplementary material for: TREM1 regulates antifungal immune responses in invasive pulmonary aspergillosis
Source: Virulence. 2021 Feb 2;12(1):570–83. doi: 10.1080/21505594.2021.1879471 (PMC7872058; doi:10.1080/21505594.2021.1879471)
Supplement: Supplemental Material [file KVIR_A_1879471_SM6548.zip › Supplementary information/Supplementary_methods.docx]

**Supplementary Methods**

**Flow cytometry**

Lung preparations were analyzed on 2, 3 or 5 dpi. Single cell suspensions were prepared in staining buffer (2.5% Foetal Calf’s Serum) in Dulbecco’s Phosphate buffered saline (BioWhittaker, Lonza Group, Visp, Switzerland) and non-specific binding was blocked with anti-Fc-Block (BD Biosciences, San Jose, CA, USA). Cell subpopulations were defined by flow cytometry techniques with the antibodies listed in Table S1 and fixed in paraformaldehyde 2%. Doublets were discriminated using the FSC-H versus FSC-W strategy, and cell viability was assessed by staining with Fixable LIVE/ DEAD violet-510 kit (Thermo Fisher Scientific) as a marker of fixed dead cells. The cells were analyzed on a LSR Fortessa X-20 (BD Biosciences) cytometer, using DIVA v8.0 (BD Biosciences) software packages.

**Supplemental Table 1. Antibodies used in flow cytometric studies**

| **MoAbs** | **Clone** | **Label** | **Company** |
| --- | --- | --- | --- |
| CD3 | 17A2 | FITC | BioLegend |
| CD4 | GK1.5· | APCCy7 | BioLegend |
| CD8 | 53-6.7 | PE | BioLegend |
| CD11b | M1/70 | PECy7 | BioLegend |
| CD19 | 6D5 | Violet 421 | BioLegend |
| CD45R/B220 | RA3-6B2 | PECy7 | BioLegend |
| Gr-1 | RB6-8C5 | PE | BioLegend |
| Isotype controls |  | FITC/PE/APC/  PECY7/APCCy7/Violet 421 | BD |

FITC, fluorescein isothiocyanate; PE, phycoerythrin; APC, allophycocyanin, PECy7, phycoerythrin conjugated with the tandem Cyanin 7; APCCy7, allophycocyanin conjugated with the tandem Cyanin 7.
